# Polymer Graphite Pencil Lead as a Cheap Alternative for Classic Conductive SPM Probes

**DOI:** 10.3390/nano9121756

**Published:** 2019-12-10

**Authors:** Alexandr Knápek, Dinara Sobola, Daniel Burda, Aleš Daňhel, Marwan Mousa, Vladimír Kolařík

**Affiliations:** 1 Institute of Scientific Instruments of the Czech Academy of Sciences, Královopolská 147, 612 64 Brno, Czech Republic; burda@isibrno.cz (D.B.); kolariq@isibrno.cz (V.K.); 2 Department of Physics, Faculty of Electrical Engineering and Communication, Brno University of Technology, Technická 8, 616 00 Brno, Czech Republic; sobola@vutbr.cz; 3 Central European Institute of Technology, Brno University of Technology, Technická 10, 616 00 Brno, Czech Republic; 4 Institute of Biophysics of the Czech Academy of Sciences, Královopolská 135, 612 65 Brno, Czech Republic; danhel@ibp.cz; 5 Department of Physics, Mu’tah University, Al-Karak 61710, Jordan; mmousa@mutah.edu.jo

**Keywords:** scanning tunneling microscopy, sharp tip formation, graphite

## Abstract

This paper presents polymer graphite (PG) as a novel material for the scanning tunneling microscopy (STM) probe. Conductive PG is a relatively modern nanocomposite material used for micro-pencil refills containing a polymer-based binding agent and graphite flakes. Its high conductivity and immunity against surface contamination, with a low price, make it seem like a highly suitable material for electrode manufacturing in general. For the tip production, three methods were developed and are further described in the paper. For the production, three commercially available polymer graphite rods were used. Each has been discussed in terms of performance within the tunneling microscope and within other potential applications.

## 1. Introduction

Graphite is a natural mineral and one of carbon’s allotropes that possesses a layered structure. It’s soft, malleable, and greasy to the touch [1]. Several unique properties make graphite a widely used material: production of electrodes, lubricants, fillers of plastics, neutron moderators in nuclear reactors, and many other applications [2,3]. Graphite is also used in the production of aluminum, synthetic diamonds, and in thermal protection of warheads of ballistic missiles and space modules. Classical pencil leads are made from a mixture of white clay (kaolinite) and graphite [4].

The classical pencil lead in its modern form was invented in 1794 by Nicolas Jacques Conte, a talented French scientist and inventor. Conte developed a recipe for mixing graphite with white clay and produced high-quality rods from these materials. Strength was achieved by heat treatment, at which the clay hardens [4]. Varying the proportion of the mixture made it possible to make rods of different hardness. The composition of modern leads includes polymers, which make it possible to achieve the desired combination of hardness, strength, and elasticity. Carbon itself as well as polymer graphite (PG) also show field-emission behavior as it was described in previous papers [5,6,7]. Main characteristics of polymer graphite which could be convenient for usage in scanning probe microscopy (SPM) are: heat resistance, considerable durability, resistance to mechanical and hydraulic stress, and, last but not least, corrosion resistance [8]. Also, it has been previously reported that polymer graphite contains up to 80% of sp^3^ hybridized carbon, moving its properties more towards graphene-based structure [9,10].

For our study, we have chosen a scanning tunneling microscopy (STM) to demonstrate the use of pencil lead in conductive SPM techniques [8]. Whether it’s a study of topography or electrical characteristics, the scanning probe microscopy is an integral part of modern research [11,12]. Possibilities of SPM are defined by both set-up configuration and sample-tip interface [13]. In order to achieve maximum resolution by STM, it is necessary to obtain an extremely sharp conductive tip serving as a probe scanning over a sample surface. The STM probes, which are currently used, are made traditionally of platinum-iridium alloy in proportions of 90:10 or 70:30 (Pt:Ir). This composition benefits from the chemical stability of platinum and an increased hardness. The main disadvantage of this material is its high price.

Another commonly used material is tungsten (W) which is used mainly for its high melting point, good conductivity, and a relatively easy way of creating the sharp tip. This is done mostly by incorporating an electrochemical etching method, where the etched tungsten wires dissolute in an alkaline electrolyte yielding a sharp tip by the increased tension of the electrolyte near its surface [14]. Tungsten is still quite an expensive material compared to the graphite pencil lead, even when working with the polycrystalline wire. Tungsten also tends to create surface oxide which blunts the tip and hence decreases the scanning resolution. From the above mentioned factors, the development of new probes is important for surface science because STM is a high-accuracy method for characterization of micromorphology and nanomorphology of surface, defects and fractures of surface, measurements of nano-scaled structures, and, last but not least, understanding mechanisms on the surface [15].

## 2. Materials and Methods

### 2.1. Materials

The market of pencil leads consists of several popular brands of mechanical pencils (e.g., Pilot, Erich Krause, Proff, Parker, Koh-i-Noor Hardtmuth, Stabilo, Pentel, Staedtler, Faber-Castell, Rotring, Bic, Conte, Index, Lamy, Constructor, and many others). The production of carbon pencils is spread over the world and each product is based on a special fabrication technology. For our experiments, we have used three different brands of polymer graphite rod, in particular: Koh-i-Noor, Staedtler, and Pentel Hi-polymer E.

### 2.2. Equipment and Methods of Analysis

For the analysis, several analytical methods were used, providing information based on different physical principles. Such a complementary approach allows for achieving reliable conclusions on the pencil lead’s chemical composition.

Raman spectroscopy was carried out in the inVia Spectrometer (Renishaw, Wotton-under-Edge, UK) utilizing green laser (DPSS 532 nm, P = 30 mW) and the exposure of 10 × 10 s with a 50× objective. The Raman spectra were evaluated and fitted with Lorentzian peak shapes using Fityk 1.3.1 software (Marcin Wojdyr, Warsaw, Poland). X-ray photoelectron spectroscopy (XPS) measurements were carried out with Kratos Supra with monochromatic Al-Kα source and photon energy of 1486.7 eV. The survey scans were measured within the binding energy range of 0–1200 eV with the step of 1.0 eV. The spectra that were referenced were evaluated in CasaXPS 2.3.22PR1 software (Casa Software Ltd., London, UK) which allows calculation of atomic percent and many different parameters like Full width at half maximum (FWHM), deconvolution for the peak.

The Scanning electron microscopy (SEM) and Energy Dispersive X-Ray Analysis (EDX) measurements were carried out in Tescan Lyra3 scanning electron microscope with an in-built Aztec SSD detector (Oxford Instruments, Abingdon-on-Thames, UK). The EDX spectra were obtained with accelerating voltage of 15 kV at a working distance of 9 mm, view the field of 20 µm, and spot size of 5.5 nm. The ICP-MS measurements were carried out in Agilent 7900 (Santa Clara, CA, USA) that is a quadrupole system offering high matrix tolerance, wide dynamic range, and an effective interference removal for trace elements across most typical applications.

#### 2.2.1. Raman Spectroscopy

**Figure 1:**
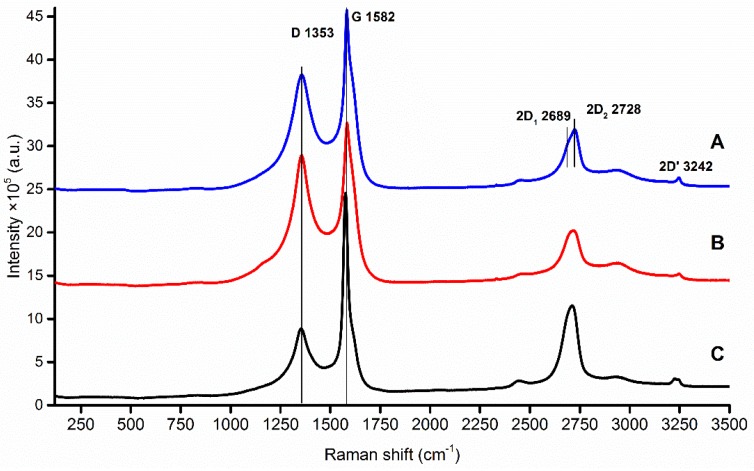
Stacked Raman spectra of each sample: (**A**) Pentel Hi-Polymer E (blue); (**B)** Koh-i-Noor (red); (**C**) STAEDTLER (black).

The Raman spectra of pencil leads show peaks common in polycrystalline graphite. The G peak was observed at 1580 cm^−1^, which arises from the bond stretching of all pairs of sp^2^ hybridized atoms; the D peak is around 1360 cm^−1^, which becomes visible in relation with the defects in sp^2^ graphite sheets, in polycrystalline graphite, and graphite-like materials with crystalline defect; the D peak overtone called 2D peak is observed at around 2690 cm^−1^, which on polycrystalline graphite samples splits into two components: 2D_1_ and 2D_2_ [16]. The above-mentioned measurements are illustrated in Figure 1.

D/G and 2D_1_/2D_2_ intensity ratios give information about the estimated size and turbostraticity of layers of polycrystalline graphite flakes, which may play a significant role in the fabrication of sharp STM tip. Properties of analysed pencil leads include Raman I_D_/I_G_ intensity ratio, calculated crystallite size, and relative atomic concentration (derived from XPS measurements) are illustrated in Table 1.

#### 2.2.2. EDX and XPS Analysis

The pencil leads were also thoroughly investigated using EDX and XPS with the aim to obtain complex information about both the surface and the bulk properties. Compositional analysis of different carbon leads was presented originally by Kariuki [17] and Navratil et al. [10].

**Figure 2:**
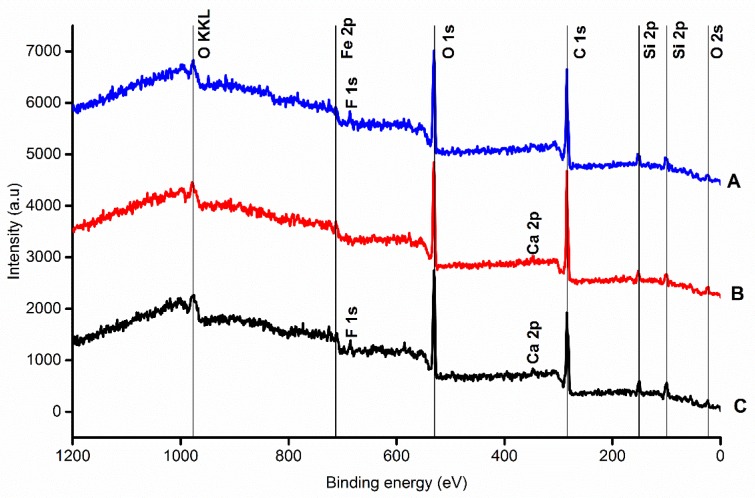
XPS spectra of each sample: (**A**) Pentel Hi-Polymer E (blue); (**B**) Koh-i-Noor (red); (**C**) Staedtler (black).

Here, we compare XPS spectra of each tip by measuring in the area where the lead has been etched in a hydroxide solution versus in the unprocessed area of the lead, yielding several double spectra for each sample presented in Figure 2. The background for the all etched areas tends to be higher than for the unprocessed areas. It was observed that Koh-i-Noor pencil leads are more stable against wet etching since the spectra before and after etching show more similarities to each other (small background for etched area, the same shape of peaks) compared to Staedtler and Pentel Hi-Polymer E leads. Furthermore, XPS analysis revealed iron contamination present at the side surface of the pencil leads, most likely due to the specific method of fabrication of those pencil leads.

**Figure 3:**
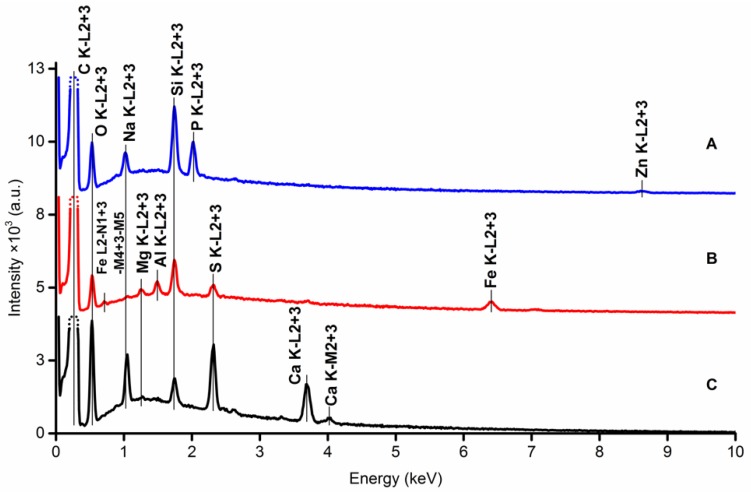
EDX measurements from the spot at the rod axis of Pentel Hi-Polymer E (**A**); Koh-i-Noor (**B**); STAEDTLER (**C**) pencil leads.

The EDX spectra were obtained from two different spots for each type of pencil lead, from the side surface of the rod and from the rod axis after breaking off a small piece of it. These measurements give valuable information about the surface and bulk; the measurements taken at the rod axis are illustrated in Figure 3. Quantitatively, Pentel Hi-Polymer E pencil lead elemental composition significantly differs from Koh-i-Noor and Staedtler leads, which both use clay-like binder containing sulfur. These findings are in good agreement with the results presented in [10].

#### 2.2.3. ICP-MS Analysis

The preparation of a sample for ICP-MS analysis was done firstly by grinding of micro-pencil rods obtaining fine-grained powder. Following this, 10 milligrams of the powder was placed in a 2.5 mL of polypropylene in an Eppendorf micro tube along with: 250 microliters of concentrated sulfuric acid (96%) containing V_2_O_5_ serving as a catalytic converter, and concentrated perchloric acid (71%), both practical grade chemicals. These suspensions were sonicated for 10 min in a laboratory ultrasound sonicator, then heated to 90 °C and shaken at 700 rpm for 30 min using an Eppendorf ThermoMixer (Eppendorf AG, Hamburg, Germany). After this procedure, the sample was diluted with 10 mL of distilled water and poured into a 20 mL syringe equipped with a nylon membrane filter (0.45 μm) from which it was injected into a 50 mL volumetric flask. After that, 3.85 mL of concentrated nitric acid (65%) was added to the volumetric flask in order to obtain a 5% solution after adding a particular volume of distilled water. Three 3 Ml samples were taken away from the solution and used for Inductively coupled plasma mass spectrometry (ICP-MS) analysis yielding 3 sets of results that were averaged. From these measurements, the arithmetic mean value and the standard deviation were calculated. The results are illustrated in Table 2.

The obtained results illustrated in the Table 2 are in agreement with the results provided by the EDX and XPS analyses. Elements like C, Cl, S, and O cannot be detected using ICP-MS. Also, phosphorus (P) and silicon (Si) also cannot be detected in our setup due the absence of particular measurement standards. Between particular brands, there is a significant difference in chemical composition of the contents, in particular the elements: Na, Li, Mg, Fe, and Zn, which is, in our opinion, caused by a specific composition of a bonding agent for each brand.

From the electrochemical point of view, Staedtler seems to be most suitable since the majority of the components are more electrochemically inert; in particular, we are referring to the elements: Na, Mg, Ca, and Fein opposition to the Koh-i-Noor brand that contains a high amount of heavy metals (Al, Ti, Cr, Mn, Co, Ni, Cu, As, and Bi) which are often more electrochemically active (i.e., whose cations are reducible at low potentials, starting at 0–1.2 V).

### 2.3. Tip Aroduction

#### 2.3.1. Electrochemical Etching

The basic method that has been tested for preparation of the graphite sharp tip is based on electrochemical etching, as it was published earlier [14]. This method is well known and also used for field-emission microscope production. The electrochemical method of tip preparation and sharpening provides good reproducibility of the tip shape and sharpness. Type and concentration of hydroxide (NaOH, KOH) allow to control the tip quality. An electrochemical etching station for probe production of NT-MDT Company (Moscow, Russia) was used. The device was partially modified by adding a precise clamp-holder to fix the graphite rod during etching. The potential between two electrodes (one is a graphite rod and the other is a metal ring) is 12 V with alternative current.

**Figure 4:**
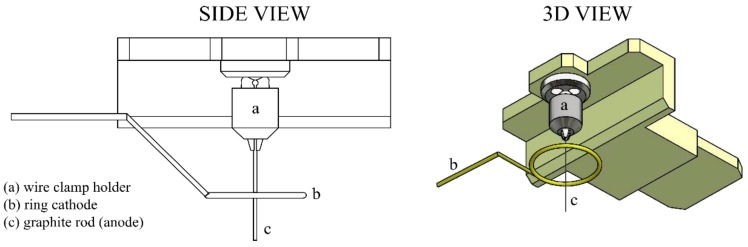
Precise clamp holder extension intended for 0.2–0.4 mm diameter wires providing precisely perpendicular fixation towards the electrolyte surface.

This allows a precisely perpendicular attachment of the tip towards the electrolyte surface, as illustrated in Figure 4, and hence increases the symmetry of the produced tip. During the etching, one of the graphite rod ends passes through a conducting diaphragm that keeps a drop of alkali solution, providing necessary surface tension cutting the rod as follows. As the bottom part falls down due to its own weight, the electric circuit providing the etching current switches off. The main requirement for adjustment of the existing setup for etching and sharping of graphite probes was the positioning of the pencil lead perpendicular to the ring electrode.

**Figure 5:**
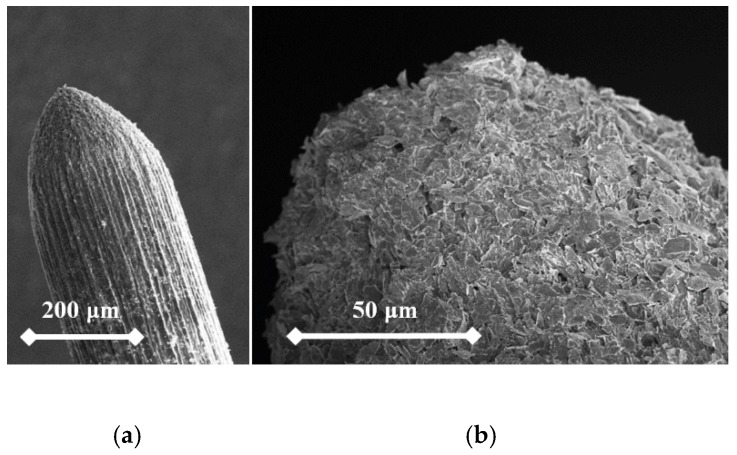
SEM image of the tip produced by electrochemical etching (**a**) and the tip surface detail (**b**) showing graphite flakes. Material used for this tip is Pentel Hi-Polymer 0.3 HB.

**Figure 6:**
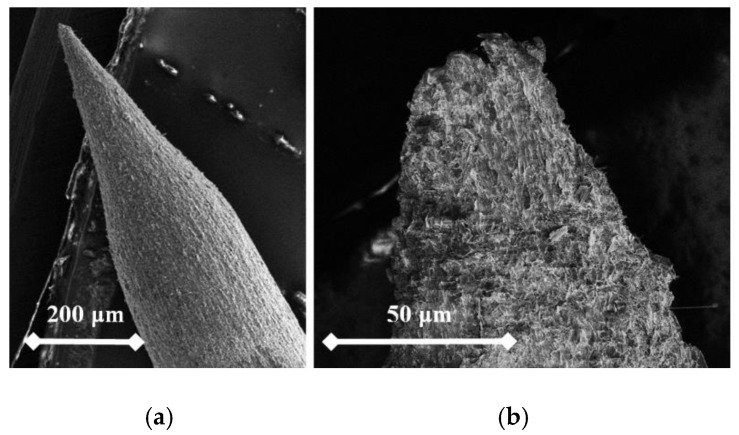
SEM image of the produced tip by electrochemical etching (**a**) and the tip surface detail (**b**) showing graphite flakes. Material used for this tip is Koh-i-Noor 0.3 HB.

**Figure 7:**
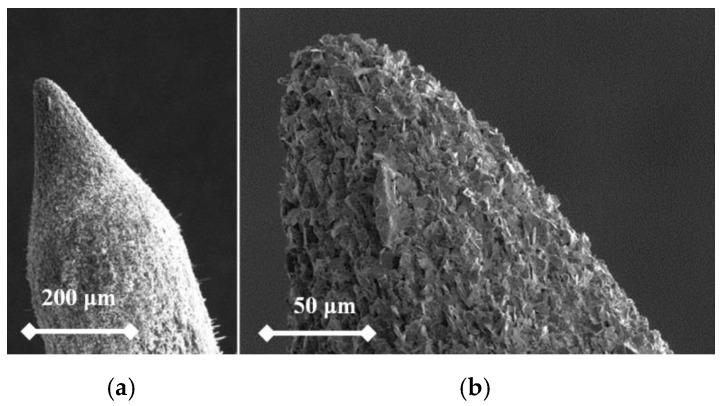
SEM image of the tip produced by electrochemical etching (**a**) and the tip surface detail (**b**) showing graphite flakes. Material used for this tip is Koh-i-Noor 0.3 HB.

From the following figures, it can be seen that for each material the produced tip is of a different shape based on different materials of the used rod. The tips were made of three different micropencil brands: Pentel Hi-Polymer 0.3 HB (Figure 5), Koh-i-Noor 0.3 HB (Figure 6), and Staedtler 0.3 HB (Figure 7). In the left part of each figure, the overall view of the tip is shown. The surface detail is illustrated in a right part of image.

#### 2.3.2. Mechanical Sharping

**Figure 8:**
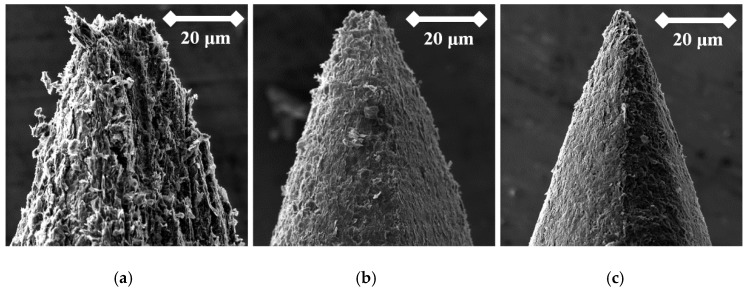
SEM image of the tip produced by mechanical sharping from the Hi-Polymer rod (**a**); by the mechanical sharping from Koh-i-Noor rod (**b**); and the mechanical sharping from Staedtler rod (**c**).

Mechanical sharping achieved by sanding is the simplest method. The only disadvantage is the low reproduciblity of the probe’s shape and the absence of a tip-sharping control (Figure 8); however, the tip sharpness may be sufficient for measurements performed in atmospheric pressure and just for preliminary control of large area samples with a large range of heights and valleys. The sharping could be carried out by grinding, for example, on sandpaper or by forming with a sharpener.

#### 2.3.3. Focused Ion Beam Milling

**Figure 9:**
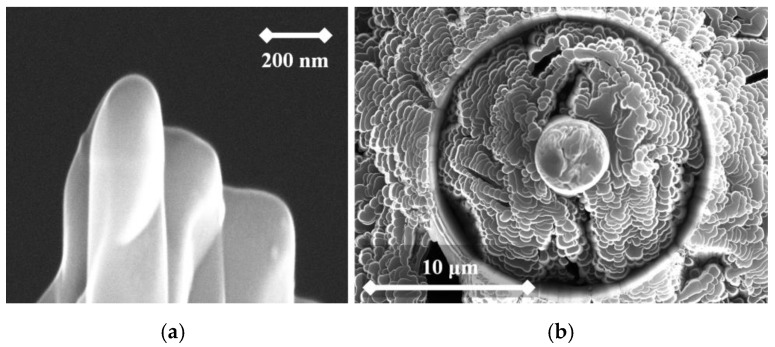
SEM image of the tip produced by FIB milling (**a**) and the tip surface detail (**b**) showing graphite flakes from top. Material used for this tip is Pentel Hi-Polymer 0.3 HB.

**Figure 10:**
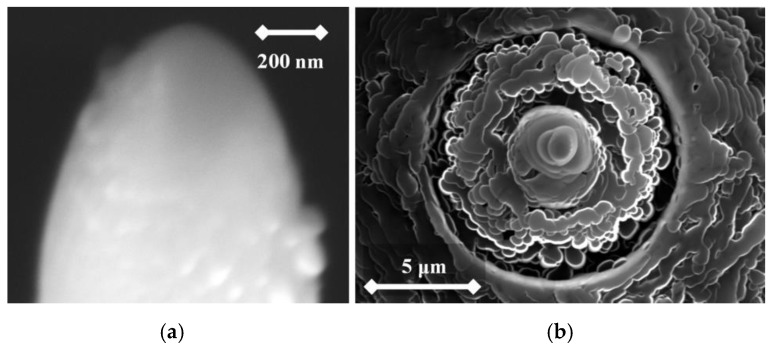
SEM image of the tip produced by FIB milling (**a**) and the tip surface detail (**b**) showing graphite flakes from top. Material used for this tip is Koh-i-Noor 0.3 HB.

**Figure 11:**
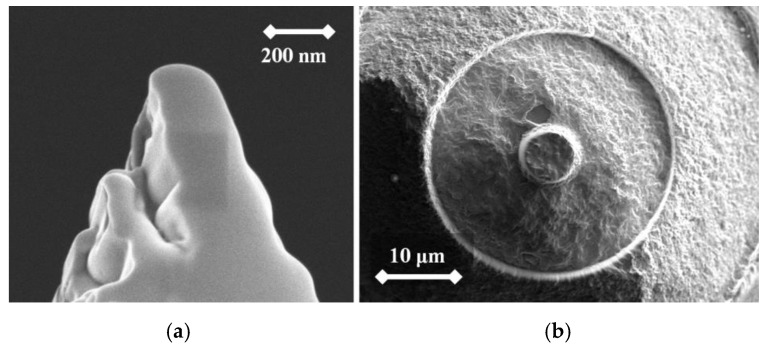
SEM image of the produced by FIB milling (**a**) and tip surface detail (**b**) showing graphite flakes from top before milling. Material used for this tip is STAEDTLER 0.3 HB.

The probes were prepared by Focused Ion Beam (FIB) at microscope Helios (FEI production, Hillsboro, OR, USA). The advantage of this microscope is the possibility of the automation of tip sharpening and the preparation of tips with desired shape. FIB processing provides control of shape and sharpness of the probes (Figure 9, Figure 10 and Figure 11). The disadvantage of this process is a high price of the final product. We used preliminarily mechanically sharpened probes to decrease the time of milling.

## 3. Results and Discussion

The results published in this paper are partially based on preliminary results that were obtained in our previous paper which discusses field emission behavior of an ultra-sharp polymer-graphite tip [7]. Electrical measurements proved that the graphite flakes behave like metallic conductors while the bonding agent used for bonding the graphite flakes within the polymer-graphite compound behaves more like an electron trap affecting current stability of the total emission current. However, in the region of tunneling currents, the emission showed quasi-stable behavior.

To test functionality, three types of probes for SPM measurements were used: the tips prepared by electrochemical etching (Figure 5, Figure 6 and Figure 7), the tips prepared by mechanical sharping (Figure 8), and the tips prepared by FIB milling (Figure 9, Figure 10 and Figure 11). Each of the tips were successfully used for SPM images acquisition. The sharpness of all our tips proved to be sufficient; however, the sharpness depended strongly on the brand of the chosen pencil lead. The lowest sharpness was obtained with Pentel HiPolymer rod. Koh-i-Noor and Stadler allow preparation of tips with small cultivate radius. FIB processing and chemical etching of the tips ensure reliable obtaining of the tips with demand geometry. Nevertheless, even the mechanical sharping proved to be sufficient during STM in air when sub-nanometer resolution is not demanded. The probes are also stable against oxidation and can be repeatedly sharpened for continuous measurement in a simple sharping procedures. The shape of the tip apex is usually created by a single flake of graphite and determines the output quality of SPM, while the precise shape of the tip does not play a significant role.

**Figure 12:**
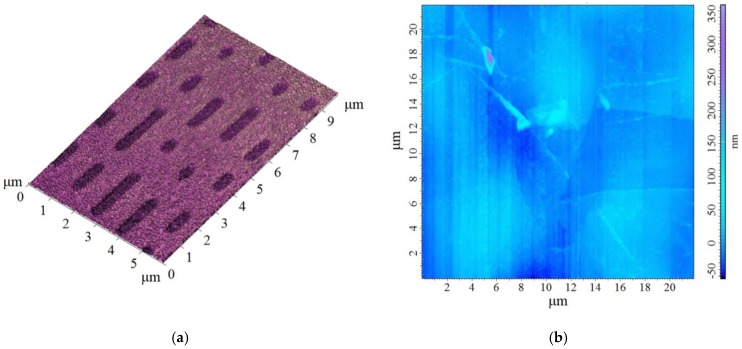
Scanning probe microscopy (SPM) image of the reference sample obtained by polymer graphite (PG) tip: (**a**) the surface of a compact disc; (**b**) the surface of a highly oriented pyrolytic graphite (HOPG).

In order to demonstrate the simplicity of working with PG probes, all the results presented were obtained using mechanically sharpened tips that can be prepared almost effortlessly just by sharpening a PG rod using sandpaper of high granularity (>1600 grains·cm^−2^). The probes were tested within an SPM NT-MDT Nanoeducator II (Moscow, Russia). As a reference sample for estimation of our probes, a surface of a compact disc (CD) and highly oriented pyrolytic graphite (HOPG) were chosen (Figure 12a). The HOPG surface (Figure 12b) was used as a reference sample for different types of electrical characterization in probe microscopy [8].

**Figure 13:**
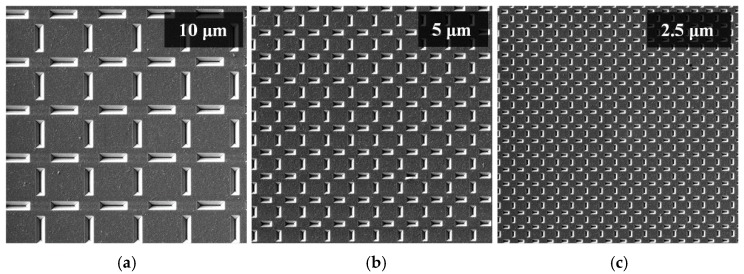
SEM image of the special calibration sample showing various grid sizes: (**a**) 10 μm grid; (**b**) 5 μm grid; (**c**) 2.5 μm grid.

In order to demonstrate the tip’s performance on a regular-structured surface, a special calibration sample that is prepared by electron-beam lithography and intended for testing a scanning electron microscope’s resolution was used. The calibration sample, which is illustrated in Figure 13, contains grids of various sizes. For our measurements, we have used a 10, 5, and 2.5 μm grids to demonstrate tip’s resolution.

**Figure 14:**
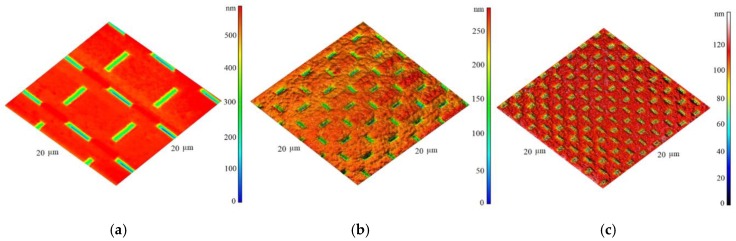
SPM image of the special calibration grid showing tip performance and spatial resolution: grid. (**a**) 10 μm grid; (**b**) 5 μm grid; (**c**) 2.5 μm

The results of the measurements are illustrated in the Figure 14 showing the three different grid sizes presented above. It can be seen that in all the grids used, the probe was able to follow the surface details precisely and also to cover a broader area without a change of the spatial sensitivity.

## 4. Conclusions

The aim of this study was to introduce different pencil lead probes for both surface visualization and modification by SPM conductive techniques. SPM is continuously developing and the methods of probe preparation and shaping are at the center of interest. Pencil lead is a cheap solution as an SPM probe for inspection of surface and its modification.

Here, we demonstrate that mechanically polished pencil leads are suitable for fast and low-resolution SPM measurements. These probes proved to be reliable for routine characterization of the samples by SPM in air, with low resolution of surface features. The cost-effective, easy preparations make them useful within the education process in laboratory SPM classes. Generally, a tip made of PG can find application in the education process in the area of scanning probe microscopy techniques or in the area of topography estimation of conductive samples with high roughness at low resolution of texture details. Electrochemical etching and FIB milling allow the preparation of probes for imaging of nanoscale surface features. Besides the introduced STM lithography by pencil lead, the probes have a potential for application at nano-grafting at atomic force microscopy techniques. The mechanical properties of flexible pencil lead could be also used for preparation in SPM techniques.

## Figures and Tables

**Table 1:** Properties of analysed pencil leads, Raman I_D_/I_G_ intensity ratio, calculated crystallite size, and relative atomic concentration derived from XPS measurements.

	Raman		EDX Contaminants		XPS [Atomic %]				
Sample	I_D_/I_G_	Crystallite Size [nm]	Side Surface	Spot at the Rod Axis	C	O	Si	Fe	F
Pentel	0.60	63	O, Na, Si, P, Fe	O, Na, Si, P, Zn	65.3	23.4	8.8	0.6	1.3
Koh-i-Noor	0.36	101	O, Na, Mg, Al, Si, P, S, Cl, K, Ca, Fe	O, Na, Mg, Al, Si, P, S, Cl, K, Ca, Fe	67.2	23.9	8.0	0.7	-
Staedtler	0.35	102	O, Na, Mg, Al, Si, S, Cl, Ca	O, Na, Si, S, Cl, Ca	48.2	24.1	15.6	0.8	1.3

**Table 2:** Results of quantitative determination of 26 elements contained in micro-pencil rods by the means of chemical dilution using acids and an additional analysis of ICP-MS.

ppm = ng·mg^−1^	Staedtler		Koh-i-Noor		Pentel Hi-Polymer E	
Element	Average (ppm)	SD	Average (ppm)	SD	Average (ppm)	SD
Li	<0.000	N/A	<0.000	N/A	404.331	5.519
Be	<0.000	N/A	<0.000	N/A	<0.000	N/A
B	<0.000	N/A	<0.000	N/A	<0.000	N/A
Na	2604.058	425.603	<0.000	N/A	<0.000	N/A
Mg	126.655	32.525	256.163	9.741	<0.000	N/A
Al	<0.000	N/A	302.484	20.995	33.291	7.363
K	<0.000	N/A	<0.000	N/A	<0.000	N/A
Ca	296.026	53.874	79.924	34.929	<0.000	N/A
Ti	2.810	0.929	49.382	5.577	3.685	1.313
V	<0.000	N/A	262.205	41.036	<0.000	N/A
Cr	7.240	0.751	5.273	0.209	5.571	0.857
Mn	10.734	1.252	43.852	0.869	2.166	1.112
Fe	168.076	48.795	4186.431	30.789	346.161	36.538
Co	<0.000	N/A	5.192	0.065	<0.000	N/A
Ni	3.978	1.266	11.485	0.380	3.979	1.023
Cu	<0.000	N/A	46.328	3.203	<0.000	N/A
Zn	<0.000	N/A	<0.000	N/A	2945.454	38.845
As	<0.000	N/A	1.079	0.144	<0.000	N/A
Se	<0.000	N/A	<0.000	N/A	<0.000	N/A
Sr	2.475	0.967	1.326	0.669	1.938	1.542
Mo	0.563	0.097	34.617	0.430	2.689	0.096
Cd	<0.000	N/A	<0.000	N/A	<0.000	N/A
Ba	3.594	1.925	6.339	1.544	73.393	2.737
Tl	<0.000	N/A	<0.000	N/A	<0.000	N/A
Pb	<0.000	N/A	<0.000	N/A	<0.000	N/A
Bi	0.451	0.033	2.405	0.026	45.076	0.585
